# 207. Ex vivo Assessment of Cefepime (FEP) Clearance during Continuous Renal Replacement Therapy (CRRT) and Clinical Validation of Optimized Dosing Regimens

**DOI:** 10.1093/ofid/ofae631.065

**Published:** 2025-01-29

**Authors:** Christina Konig, Cole McGrath, Hanna Roenfanz, Yuwei Shen, David P Nicolau, Joseph L Kuti

**Affiliations:** Hartford Hospital, Hartford, Connecticut; Hartford Hospital, Hartford, Connecticut; Hartford Hospital, Hartford, Connecticut; Hartford Hospital, Hartford, Connecticut; Hartford Hospital, Hartford, Connecticut; Hartford Hospital, Hartford, Connecticut

## Abstract

**Background:**

Optimal antibiotic dosing in critically ill patients receiving CRRT is crucial. Drug clearance (CL) can be affected by multiple factors such as CRRT mode, effluent rate (ER) or filter type. We investigated FEP transmembrane CL (CL_TM_) in an ex vivo CRRT model to inform optimal dosing regimens and validate these regimens by re-simulation in a clinical cohort of CRRT patients.

**Figure:**
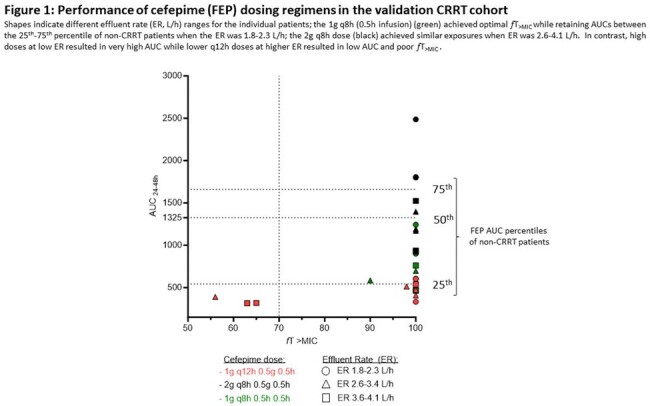

**Methods:**

CL_TM_ was determined ex vivo in hemofiltration and hemodialysis modes with M100 and HF1400 filters. Bovine blood spiked with FEP was sampled from pre-, post-filter and effluent fluids at 10, 30, and 60 minutes in duplicate runs at ER of 1, 2 and 3 L/h. CL_TM_ was calculated via sieving (SC) and saturation coefficients (SA). A multiple linear regression was performed to describe CL_TM_ as a function of ER, filter, and mode. Monte Carlo Simulations (MCS; n=1000) using the equation CL= non-renal CL + CL_TM_ and distribution parameters from a published population pharmacokinetic (popPK) model were executed for ascending ER (1-5 L/h). The resulting FEP regimens were selected by probability of target attainment (PTA) for 70%*f*T > MIC (MIC = 8mg/L CLSI susceptible breakpoint) and mean AUC within the 25^th^-75^th^ percentile of a non-CRRT cohort. Selected regimens were validated by re-simulating *f*T > MIC and AUC using popPK defined Bayesian parameters in Pmetrics for R from 10 CRRT patients receiving FEP in clinical practice.

**Results:**

Mean (SD) ex vivo SC/SA were 1.01 (0.08). ER was the main predictor (p< 0.001) for CL_TM_ (CL_TM_= 0.139+0.841*ER) with no effect from mode or filter type. MCS of FEP 1g and 2g q8h provided 100% PTA at 8 mg/L with AUCs similar to the non-CRRT comparator for ER of 1-2.4 and 2.5-5 L/h respectively. Mean (SD) popPK parameter estimates of the validation cohort were CL, 3.8 (1.1); Vc, 17.3 (15.3) L; k12, 5.0 (3.1) h^-1^; and k21, 6.4 (7.6) h^-1^. Selected regimens tested in the validation cohort demonstrated good performance with the algorithm (Figure 1).

**Conclusion:**

These are the first data to translate the findings of ex vivo CRRT CL_TM_ in a dosing algorithm based on CRRT ER to achievable exposure in patients receiving FEP. For CRRT ER of 1-2.4 L/h and 2.5-5 L/h FEP 1g and 2g 8h as 0.5h infusions achieve 70% *f*T > MIC, respectively, while retaining AUC in range with safe exposures in non-CRRT patients.

**Disclosures:**

**Christina Konig, PhD**, Gilead Inc.: Honoraria|Pfizer Inc.: Honoraria|Shionogi Inc.: Honoraria **David P. Nicolau, PharmD**, CARB-X: Grant/Research Support|Innoviva: Grant/Research Support|Innoviva: Honoraria|Merck: Advisor/Consultant|Merck: Grant/Research Support|Merck: Honoraria|Pfizer: Advisor/Consultant|Pfizer: Grant/Research Support|Pfizer: Honoraria|Shionogi: Advisor/Consultant|Shionogi: Grant/Research Support|Shionogi: Honoraria|Venatorx: Grant/Research Support **Joseph L. Kuti, PharmD**, Abbvie: Advisor/Consultant|bioMerieux: Grant/Research Support|Merck: Grant/Research Support|Pfizer: Grant/Research Support|Shionogi Inc: Advisor/Consultant|Shionogi Inc: Grant/Research Support|Shionogi Inc: Honoraria|Venatorx: Grant/Research Support

